# Morphological and Nutritional Properties of Moroccan* Capparis spinosa* Seeds

**DOI:** 10.1155/2019/8594820

**Published:** 2019-04-23

**Authors:** Nidal El amri, Faouzi Errachidi, Abdellatif Bour, Sara Bouhaddaoui, Rachida Chabir

**Affiliations:** ^1^Laboratory of Human Pathology, Biomedicine and Environment, Sidi Mohammed Ben Abdellah University, BP 30050, Fez, Morocco; ^2^Laboratory Biology and Health, Faculty of Science, Ibn Tofail University, BP 133, Kenitra, Morocco; ^3^Laboratory of Functional Ecology and Environment, Sidi Mohammed Ben Abdellah University, BP 30050, Fez, Morocco; ^4^Laboratory of Physiology, Molecular Genetics and Biotechnology, Hassan II University, BP 8110, Casablanca, Morocco

## Abstract

*Capparis spinosa* is one of the few shrub species which has so many qualities with many uses. In this case, the present work aimed to study both some morphological characteristics and biochemical components (proteins, lipids, and carotenoids) of fresh* C. spinosa* seeds with three different sizes, collected from two Moroccan regions. In this study,* C. spinosa* seeds present a total of proteins ranging from 23.32 to 28.5% on a dry weight basis. Additionally, the total lipids varied between 2.8 and 3.4%.* C. spinosa* seeds contained a high level of carotenoids. Besides, the analytical values have been variously affected by both size and location. Further, the preliminary morphological and anatomical characterization of leaves, stems, and morphological properties such as length, width, thickness, geometric mean diameter, sphericity, surface area, and mass of 100 seeds have been determined. Consequently, this present study confirms the importance of Moroccan* C. spinosa* seeds, which represent a significant nutritional value. Also, its good morphological quality is a significant indicator of commercial criteria.

## 1. Introduction

“*Capparis spinosa”* is a perennial plant that is best known for the edible flower buds. Spontaneous plants, xerophyte and heliophile, are very widespread in the Mediterranean basin [1–3] due to their tolerate climatic constraints in arid and semiarid zones as well as extreme temperatures. Therefore, they can play a very useful ecological role in these regions for protection against erosion. Also the caper is cultivated. In this respect, it provides a sought after condiment, caper, which corresponds to the floral button of the plant. Moreover, it is used as fodder as well as ornamental plant that is used in honey making. Above all, it possesses important medicinal qualities used in traditional medicine [4, 5].

The main economic utility of* Capparis spinosa* is derived from trade in its flower buds, commonly known as capers, which are the subject of considerable trade, particularly at the international level. In this case, its floral buds are particularly popular in international markets because of their use as a condiment or ornament, their consumption could increase significantly, and they could also be the subject of other interesting uses from view, especially in the cosmetics sector. In addition, the caper could easily be developed in the context of agriculture. Thus, a quality production offers the caper a great added value, which allows it a large diffusion in the international market.

In Morocco,* Capparis spinosa* is characterized by a wide geographical distribution [1, 6–8]. It grows spontaneously in the wild and its cultivation depends on origins. Besides, its culture plays a predominant role in the agricultural economy of the marginal areas in the provinces of Fez and Safi.

To our knowledge, no detailed studies concerning physical and chemical properties of Moroccan* Capparis spinosa* seeds have been performed; therefore knowledge of these physical properties is very important for industries that provide essential engineering data. Additionally, the size, shape, and physical dimensions of seeds are crucial in sizing, sorting, and separation. For this reason, the aim of this study is to determine and compare the morphological and biochemical (lipids, proteins, and carotenoids) properties of raw* Capparis spinosa* seeds (capers) collected from two regions in Morocco (FEZ and SAFI) and having different seed sizes in order to provide new basic data on the composition of Moroccan caper. Moreover, preliminary morphological and anatomical characterizations of leaves and stems have been determined.

## 2. Materials and Methods

### 2.1. Study Sites

**Figure 1:**
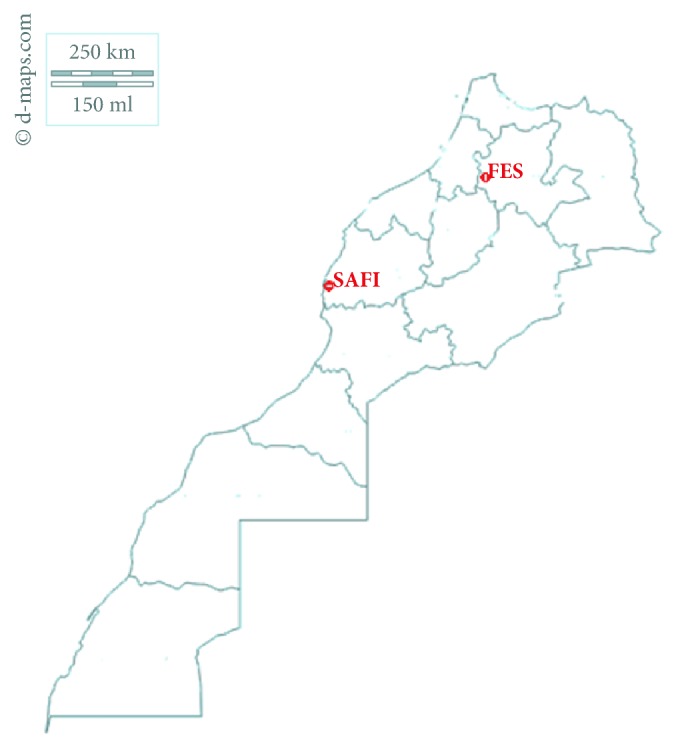
Geographic location of the tow harvested samples of* Capparis spinosa*.

Our project has been conducted in the FEZ region (34° 01′59^″^ North Latitude and 5° 00′01^″^ Longitude West) with the village of Ouled Jama located at 333 meters (34° 13′48^″^N and 5° 4′48^″^ W) and the region of SAFI located 45 meters above sea level (32° 17. 9634′ North Latitude, 9° 14. 2308′ Longitude: West) with the village Sebt Gzoula (Latitude: 32 07′ 00′′ latitude North (-9 05′ 00′′ West longitude) (Figure 1).

In the Safi region (semiarid climate with warm winter),* Capparis spinosa* normally grows on wind-prone hills for several months of the year. Besides, it is also found on light sandy loam soils with alkaline pH (6 to 9). In FEZ region (semiarid climate with temperate winter), it is found on Hamri soil clay and poorly draining.

### 2.2. Plant Material

This study concerns the genus* Capparis* sp.* “Capparis spinosa”* cultivated in SAFI and FEZ regions, collected in July 2016. From each region, stem, leaves, and seeds of* Capparis spinosa* plant have been collected. In addition, the raw seeds have been collected from 12 plants and mixed and then they have been classified in three different sizes (diameter): ≥7mm, 8/9 mm, and 11/12 mm.

### 2.3. Morphological and Anatomical Analysis of Leaves and Stems

Three caper plants have been chosen from each region. To add, samples of 90 leaves and 9 stems distributed over the three selected plants (30 leaves and 3 stems per plant) have been taken for the characterization of each region. Thus, four characters have been described and divided into classes: shape and color of the leaf, shape and color of the stems, and shape and color of the spines. Also, four other quantitative traits have been measured: length and width of the leaf, petiole length, and number of ribs leaves.

For anatomical studies, fresh samples of stems have been cross-sectioned by hand. In addition, the slide preparations have been stained independently with green carmine-iodine and then observed under a petrographic microscope (Olympus BX40). Microphotographs have been taken using a digital camera (Ikegami) focused through the microscope eyepiece.

### 2.4. Morphological Parameters of Seeds

The* Capparis spinosa* seeds have been manually cleaned to remove all foreign and broken seeds. In order to determine the physical properties of* Capparis spinosa* seeds, the length, width, and thickness of 100 random seeds (capers) have been measured using a micrometer with an accuracy of 0.01 mm. The mass of 100 seeds has been weighed by a precision numerical balance.

The geometric mean diameter Dg of the seed has been calculated by using the following formula [9]:

(1) $$\begin{array}{c} Dg={\left(\;L\times W\times T\;\right)}^{1/3} \end{array}$$

where L is the length, W is the width, and T is the thickness.

The sphericity Ø of seeds has been determined by the ted using the following relationship [9]:

(2) $$\begin{array}{c} Ø=\frac{{\left(L\times W\times T\right)}^{1/3}}{L} \end{array}$$

The surface area S in mm of caper seed has been found by analogy with a sphere of same geometric mean diameter, using the following relationship [10–12]:

(3) $$\begin{array}{c} S=\pi {rDg}^{2} \end{array}$$

### 2.5. Nutritional Properties

*The total lipid* content has been determined according to ISO method 659:1998 [13]. 10 g of* C. spinosa* seeds has been ground in a mortar and extracted with hexane in a Soxhlet apparatus for 4 h; the solvent has been concentrated using a rotary evaporator under reduced pressure at 50°C.

*The total carotenoid* content of the samples has been determined spectrophotometrically according to Sumanta et al. (2014) [14]; briefly, 0.5 g of caper has been successively extracted with 10 ml of acetone 80%. This homogenate has been centrifuged for 14,000 RPM for 10min at 4°C. Besides, the supernatant has been separated and 0.5 ml of it is mixed with 4.5 ml of acetone solvent. Moreover, the solution mixture has been analyzed for carotenoid content in spectrophotometer. The following equation has been used to calculate total carotenoids [14]:

(4) Ch−a=12.25A663.2−279A646.8

(5) Ch−b=21.5A646.8−5.1 A663.2

(6) C=1000A470−1.82Cha−85.02Chb198

*The protein content* of the samples has been determined by Nasri et al. (2007) [15], Tlili et al. (2011) [16], and Biuret methods. Shortly, 0.5g of caper has been extracted by the alkaline solution containing 50 mmol Tris–HCl, 200 mmol NaCl, SDS, pH 8.5, and centrifuged (14,000g for 15 min). 4 ml of Biuret reagent has been added to 1ml of protein extract. The absorbance has been measured at 540 nm. BSA has been used as the standard for the calibration curve (0 to 1g/l). Finally, the rest of the biochemical properties of seeds have been analyzed according to AOAC (1984) [17].

### 2.6. Isolation of the Essential Oil

The sample has been ground and then extracted by hydrodistillation using a Clevenger type apparatus: the extraction lasted 5h30′ by placing 200 g of dry leaves in a flask with distilled water and then heating; the vapors are condensed in a condenser and the oils are separated from water by difference in density. The yield is defined as the ratio between the mass of the essential oil obtained and the mass of plant material to be treated.

### 2.7. Statistical Analysis

Experiments and analyses have been conducted with three replications. Besides, statistical analysis has been done by analysis of variance (ANOVA). P ≤ 0.05 has been considered to be statistically significant by Duncan's new multiple range test.

## 3. Results and Discussion

### 3.1. Morphological and Anatomical Characterization of Leaves and Stems

**Figure 2:**
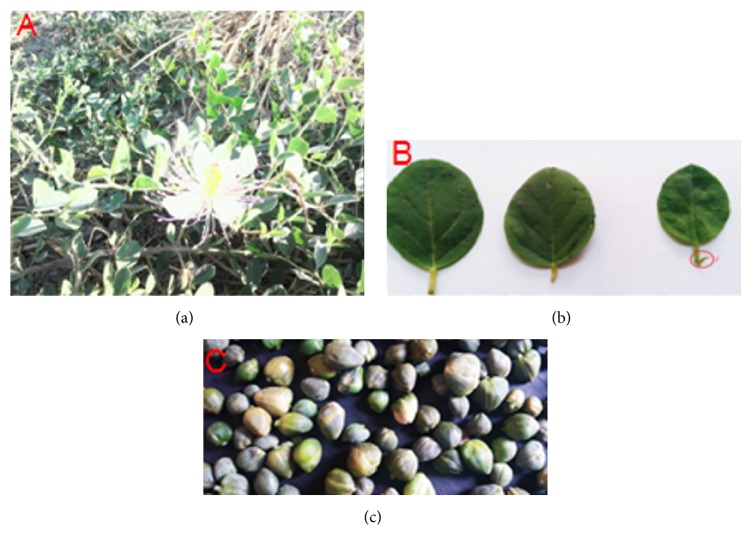
Different parts of* Capparis spinosa* studied: (a) plant, (b) leaves, (c) and seeds.

The morphological observations have revealed that studied leaves of* Capparis spinosa* have been alternated and oval-rounded; these leaves had at the base of their petiole 2 curved spines, which are short, crooked, and vulnerable (Figure 2(b)). To add, they were light green leaves with very apparent veins. Further, stems were green to red-purple, thickset, and short, and the stems became swollen near to the ground.

Measurement of the quantitative characteristics has revealed that the length of the leaves ranged between 19 and 42 mm (with an average of 30.81 ± 1,215 in FEZ and between 19 and 40 mm (with an average of 29.46 ± 0,451) in Safi, while their width ranged between 15 and 30 mm in FES and between 19 and 40 mm for SAFI. Petiole length ranged from 3 to 9 mm for Safi and from 3 to 8 mm for Safi (Table 1). The stipule of the leaves has been modified in spines to adapt to their habitat. The spines could reach 2-5 mm in length of a yellow/orange color, generally oriented downwards (Part I, Figure 2(b)).

**Figure 3:**
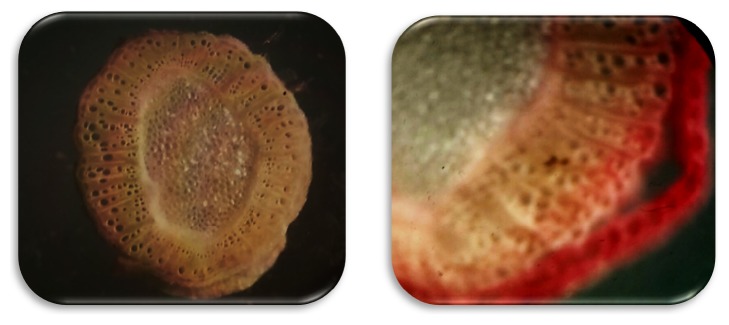
Anatomical structure of a* Capparis spinosa *stem (40X).

The outcomes of cross-sections of the* C. spinosa* stem have shown important characteristics. The epidermis had cells and the distinct cortex area had small cells. In addition, the xylem of the stem has been extremely well developed, porous to semiporous annals as shown in Figure 3. Further, the fibrovascular system in the stems and inflated transfer regions have been well evolved to maintain satisfactory water balance during periods of drought. For this reason, all these characteristics improve the adaptability of* C. spinosa* and allow it to grow and survive in extremely dry and arid conditions [18]

### 3.2. Morphological Properties of Seeds

Table 2 displays the different morphological parameters studied. The variance analysis of the parameters, length, width, thickness, weight, geometric mean diameter, sphericity, and surface area, showed a significant difference between the two populations. Generally, the mean size of 100 beads from the two regions varies between 8.65 mm and 7.97 mm, 6.03 mm, 7.44 mm, 0.87 mm, and 1.84 cm for length, width, thickness, geometric mean diameter, sphericity, and surface area, respectively, and 7.89 mm length, 7.44 mm width, 5.34 mm thicknesses, 6.77 mm geometric mean diameter, 0.86 mm sphericity, and 1.51 cm surface area. The width/length of seeds increases in parallel with the size. Our results also exhibited a crucial difference in weight and the population with the highest weight was FEZ (13.7 g). The low weight of the seeds results from small dimensions.

**Figure 4:**
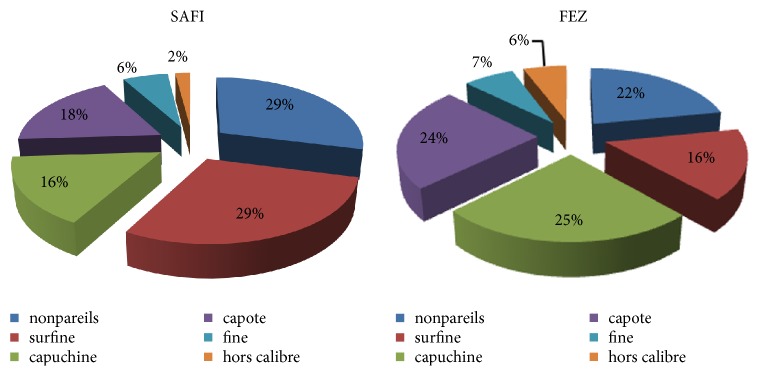
Calibers of Moroccan* Capparis spinosa* seeds collected from FEZ and SAFI.

Smaller sizes are also desired to obtain quality products due to the processing without opening. For this reason, the capers have been commercially divided into 5 groups: Nonpareils (7 mm), Surfines (7/8 mm), Capuchins (8/9 mm), Capotes (9/11), Seconds (11/12), and Hors calibers (≥ 13) [19]. In this study, we have found that the very small seeds account for more than 60% of the total capers collected (SAFI region) and 29%, 29%, 18%, 16%, 6%, and 2% of Nonpareils, surfing, Capuchins, Capotes, Seconds and Hors calibers, respectively (Figure 4). Compared to other studies, the morphological characteristics of* Capparis spinosa* seeds differ from one variety to another and from one region to another. The morphological parameters of Turkish* Capparis spinosa* studied by Özcan et al. (2004) [20] included 10.34 mm for length, 9.21 mm for diameter, and 0.925 mm for sphericity and those published by Ulukapi et al. (2016) [21] were 9.19 mm for length, 7.51 mm for width, 9.78 mm for thickness, 8.74 mm for geometric mean diameter, and 0.9 mm for surface area. In this study, it has been seen that dimensional properties of Moroccan* C. spinosa* were lower compared to other references [12, 21–23]. In this case, these differences could be the result of both genetic and environmental factors and growth conditions. Also, it is considered to be important because of their nutritive, physiological, and technological significance.

The morphological properties of caper seeds are necessary for the design of equipment to handle collection, transport, processing, and storage of the produces. In this respect, several recent studies have revealed that dimensions of caper are quite important due to buds' area of usage (food, animal feeding, cosmetics, animal feeding, etc.). The late collecting process widens bud volume but decreases biochemical and nutritional content. The outcomes of this present study show that the Moroccan* Capparis spinosa* has morphological characteristics allowing it to be positioned as a product of superior quality very desirable by the industries.

### 3.3. Nutritional Properties

The results in Table 3 show the some chemical values of three different sizes of* C. spinosa* seeds from two different locations in Morocco (FEZ and SAFI region). Water (76.8 - 81.6%), crude oil (2.7 - 3.4%), and reducing sugar (4.87 - 6.45%) values have been augmented by the size of* Capparis spinosa* seeds. These outcomes were in agreement with the study published by Özcan et al. (2004) and Arslan et al. (2007) [20, 24] who reported that crude protein decreased in size and water and crude oil and reducing sugars have been increased in size of caper. Also the values found in our study were significantly higher than the values reported by Özcan et al. (2004) [20], except for water. Additionally, our results were also in agreement with the study published by Sessiz et al. (2007) [12] who reported that length, width, thickness, geometric mean diameter, and sphericity of seeds have been increased linearly with the increase in moisture. In the study conducted by Ulukapi et al. (2016) [21] who worked on biochemical and dimensional properties of naturally grown* Capparis spinosa, *they found that* C. spinosa seeds *were rich in glucose (8113.42 mg/Kg), saccharose (14546.0 mg/Kg), and fructose (3653.07 mg/Kg), which encourages its use for animal feeding.

The* lipid content* determined in our study varies between 2.7 and 3.4 %. This value was not affected by location, but the highest crude lipid was found in big seeds. These outcomes are lower than that found by Tlili et al. (2009, 2010) [25, 26], while they were higher than those published by Özcan and Akgul (1998) [22] and Özcan et al. (2004) [20] who reported that the Turkish capers (*Capparis spinosa*) contained 1.65 and 1.35 ± 0.04% of crude oil, respectively. These differences could be due to geographic distribution, soil, climatic conditions of an area, and size of seeds or/and using different analysis methods [20, 27]. In another study carried out by Mattus and Özcan (2005) [28], the composition of fatty acids, tocopherols, and sterols of* Capparis spinosa* oil seeds has been characterized. These authors clarified that the oil contains linoleic acid as the major fatty acid accompanied by oleic acid (24.6-50.5%) and its isomer vaccenic acid (10 and 30%). The seed oils were also rich in tocopherols with the following composition: *γ*-tocopherol, 124.3-1944.9 mg/100 g; *δ*-tocopherol, 2.7-269.5 mg/100 g; and R-tocopherol, 0.6-13.8 mg/100 g. In line with this, several studies [29–33], have indicated that* Capparis spinosa* oil has also rich amount of minerals (Ca, B, Cr, Cu, K, Mg, Mn, P, S, Fe, and Zn).

The* essential oil yield* obtained ranged between 0.078% and 0.08% and this result was similar to that published by Özcan et al. (2004) [20], while our values are lower than those found by El-Ghorab et al. (2007) [34] who reported that the yield of oils was 0.30 ± 0.1% from caper buds and 0.20 ± 0.0% from caper leaves obtained from the samples with steam distillation followed by dichloromethane extraction; these authors also reported that the major volatile compounds found in caper bud oil were benzyl alcohol (20.4%), furfural (7.4%), ethanal methyl pentyl acetal (5.9%), 4-vinyl guaiacol (5.3%), thymol (5.1%), octanoic acid (4.8%), and methyl isothiocyanate (4.5%).

**Figure 5:**
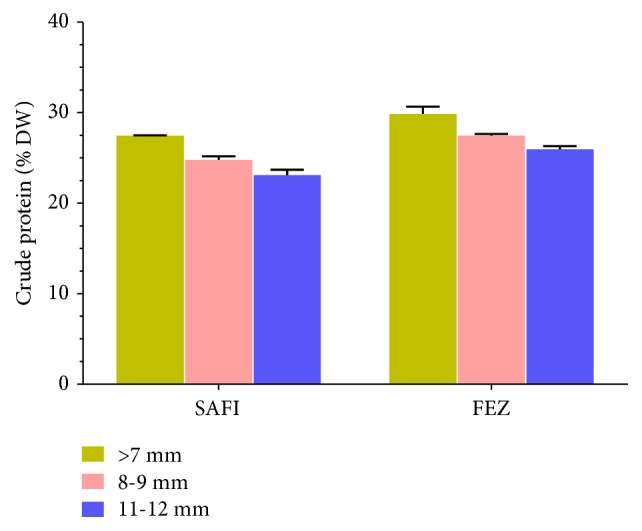
Protein content of three different sizes of Moroccan* Capparis spinosa* seeds collected from FEZ and SAFI.

The* protein contents* of three different sizes are represented in Figure 5. The protein content varies between 23.1 and 29.8 % on a dry weight basis (DW %). In general, the highest crude protein has been found in small seeds and it is higher in Fez's* C. spinosa* seeds. These results were consistent with those published by Tlili et al. (2011) [16] and Giuffrida et al. (2002) [35]. While we found higher protein content than Turkish capers (22%) studied by Akgul and Özcan (1999) [36], we found a significant difference between populations. This difference was probably due to geographic distribution, which is in agreement with other studies [15, 37].

As macronutrients, protein is an essential part of the human diet, especially in developing countries, where the average protein is less than required [38]; vegetable proteins remain a very important source for food and feed. The results showed that* C. spinosa* seeds (ca. 27%) can be used as a new source of vegetable proteins.

**Figure 6:**
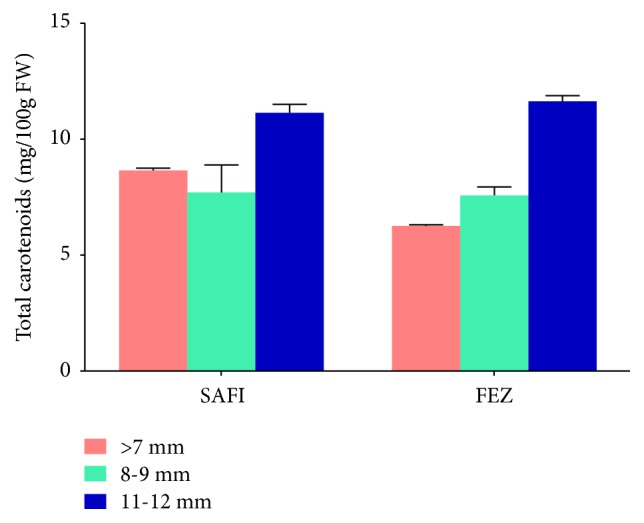
Carotenoids content of three different sizes of Moroccan* Capparis spinosa* seeds collected from FEZ and SAFI.

As presented in Figure 6, the content of total carotenoids of* C. spinosa* seed ranged between 6.26 and 11.63 (FEZ) and between 8.64 and 11.13mg/100 g (SAFI). Results displayed that big seeds contained more of total carotenoids than the smaller size, which is in agreement with the study reported by Özcan and Akgul (1998) [22]. These results demonstrate that seeds of* C. spinosa* contained significant concentrations of carotenoids (6.96 - 17.5 mg/Kg). Similar findings have also been published by Tlili et al. (2010) [39] who reported that the total carotenoid constituents of leaves and flower buds from Tunisian* C. spinosa* ranged between 18.52 and 4.83 mg/100 g FW, respectively. These authors reported a high content of lutein (ca. 8 and 2.2 mg/100 g, for leaves and seeds, resp.) and *β*-carotene (ca. 5 and 1.1 mg/100 g, for leaves and seeds, resp.).

Carotenoids play an important role in human health. The beneficial effects derived from these compounds have been attributed to their antioxidant activity, protecting cells, and tissues from the harmful effects of free radicals and singlet oxygen. Carotenoids also play a preventive role against a number of degenerative diseases and can be used in many personal care products [40]. The richness of the seeds of* C. spinosa* in carotenoids makes it a good source of these natural compounds.

## 4. Conclusion 

In this study, both morphological properties and nutritional values of Moroccan* C. spinosa* seeds naturally grown in Morocco Mediterranean climate have been investigated. In this case, results show an important level of protein (ca. 27%) in* C. spinosa* seed and high total lipid content (ca. 3.3%). In addition, reducing sugars and crude protein content of small bud size have been found to be higher than those in other sizes; however, crude oil, carotenoids, and water were higher in big seeds. Moreover,* C. spinosa* seeds are a crucial source of carotenoids. In conclusion, the morphological properties and chemical compositions of capers have been affected by size and location.

## Figures and Tables

**Table 1:** Morphometric characterization of *Capparis spinosa* leaves collected from FEZ and SAFI.

	FEZ	SAFI
Length, mm	30.81 ± 1.215^a^	29.46 ± 0.451^b^
Width, mm	22.397 ± 0.438^a^	20.633 ± 0.354^b^
Number of petioles	5.353 ± 0.719^a^	4.497 ± 0.505^b^
Number of ribs	11.3 ± 0.3	10.5 ± 0.25

Values are mean ±standard deviation.

Different letters indicate significant differences at p≤ 0.05.

**Table 2:** Morphological properties of *Capparis spinosa* seeds.

	FEZ	SAFI
Length, mm	8.65 ± 0.16^a^	7.89 ± 0.11^b^

Width, mm	7.97 ± 0.34^a^	7.44 ± 0.22^b^

Thickness, mm	6.03 ± 0.2	5.34 ± 0.12

Width/length of seeds	0.92	0.93

Geometric mean diameter (Dg) mm	7.44^a^	6.77^b^

Sphericity (Q), mm	0.87	0.86

Surface area (S), cm	1.84	1.51

One hundred seeds mass, g	13.7	12.3

Test F. Each value is the mean ± Standard deviation.

Different letters indicate significant differences at p≤ 0.05.

**Table 3:** Proximate composition of three different sizes of *Capparis spinosa* seeds from FEZ and SAFI.

	Water %			Reducing sugars (%DW)			Crude lipid (%)		
	Seed size (Diameter mm)								
	≥7	8-9	11-12	≥7	8-9	11-12	≥7	8-9	11-12
SAFI	76.8 ± 0.4^a^	77.82 ±0.56^b^	79.51± 0.3^C^	6.45 ± 0.36^a^	5.69 ± 0.011^b^	4.87 ± 0.005^c^	2.8 ± 0.42^a^	2.90 ±0..22^a^	3.4±0.4^b^

FEZ	78.04 ± 0.23^d^	80.9 ±0.7^e^	81.6 ± 0.52^f^	5.86 ± 0.017^b^	4.99 ± 0.01^c^	4.97 ± 0.005^c^	2.72 ± 0.4^a^	2.91± 0.3^a^	3.3 ±0.1^b^

Test F. Each value is the mean ± Standard deviation.

Different letters indicate significant differences at p≤ 0.05.

## Data Availability

The data used to support the findings of this study are included within the article. They represent an average analysis of three repetitions and are present in tables with their standard deviation.
